# Enhanced Mechanical Properties of Ti/Mg Laminated Composites Using a Differential Temperature Rolling Process under a Protective Atmosphere

**DOI:** 10.3390/ma17112753

**Published:** 2024-06-05

**Authors:** Zichen Qi, Zhengchi Jia, Xiaoqing Wen, Hong Xiao, Xiao Liu, Dawei Gu, Bo Chen, Xujian Jiang

**Affiliations:** 1 College of Mechanical Engineering, Zhejiang University of Technology, Hangzhou 310023, China; qizichen@zjut.edu.cn (Z.Q.); 2112102229@zjut.edu.cn (Z.J.); goodavid@zjut.edu.cn (D.G.); jiangxujian1519@163.com (X.J.); 2 National Engineering Research Center for Equipment and Technology of Cold Strip Rolling, Yanshan University, Qinhuangdao 066004, China; 3 Zhejiang YaTong Advanced Materials Co., Ltd., Hangzhou 310030, China; wenxiaoqing1992@163.com; 4 College of Mechanical and Vehicle Engineering, Taiyuan University of Technology, Taiyuan 030024, China; liuxiao@tyut.edu.cn

**Keywords:** Ti/Mg composite plates, induction heating, protective atmosphere, differential temperature rolling, bonding strength

## Abstract

Addressing the issue of low bonding strength in Ti/Mg laminated composites due to interfacial oxidation, this study employs a differential temperature rolling method using longitudinal induction heating to fabricate Ti/Mg composite plates. The entire process is conducted under an argon gas protective atmosphere, which prevents interfacial oxidation while achieving uniform deformation. The effects of reduction on the mechanical properties and microstructure of the composite plates are thoroughly investigated. Results indicate that as the reduction increases, the bonding strength gradually increases, mainly attributed to the increased mechanical interlocking area and a broader element diffusion layer. This corresponds to a transition from a brittle to a ductile fracture at the microscopic tensile–shear fracture surface. When the reduction reaches 47.5%, the Ti/Mg interfacial strength reaches 63 MPa, which is approximately a 20% improvement compared to the bonded strength with previous oxidation at the interface. Notably, at a low reduction of 17.5%, the bonding strength is significantly enhanced by about one time. Additionally, it was found that a strong bonded interface at a high reduction is beneficial in hindering the propagation of interfacial cracks during tensile testing, enhancing the ability of the Ti/Mg composite plates to resist interfacial delamination.

## 1. Introduction

Magnesium alloys are the lightest structural metals with a density two-thirds that of aluminum alloys, offering high specific strength and specific stiffness, among other superior properties that make them highly promising materials for automotive and aerospace applications [1,2,3,4]. However, the poor corrosion resistance of magnesium alloys has limited their development [5,6,7]. Therefore, it is crucial to form bimetal plates with other corrosion-resistant metals. Titanium and titanium alloys, as new structural materials, are widely used in the aerospace industry for their low density, high strength, corrosion resistance, wear resistance, and high-temperature impact resistance [8,9,10]. Utilizing the lightweight nature of magnesium alloys and the excellent corrosion resistance of titanium and titanium alloys, the development of new lightweight Ti/Mg laminated composites has broad application prospects [11,12,13,14].

Preparation methods for Ti/Mg composite plates mainly include explosive welding, diffusion welding, explosive + rolling, and hot rolling composite methods. Wu et al. [15] successfully prepared Ti/Mg composite plates through explosive welding, achieving a maximum bonding strength of 64 MPa, and studied the mechanical properties and interfacial bonding mechanisms of the composite plates. However, the explosive welding process generates seismic waves, noise, and toxic gases, which are not conducive to the large-scale production of composite plates. Tan et al. [16] used an AZ91 magnesium-based brazing wire as an intermediate material to achieve a continuously uniform welding joint between magnesium alloy and titanium alloy through laser brazing. Xiong Jiangtao et al. [17] used aluminum foil as an intermediate layer for transient liquid phase diffusion welding of magnesium alloy and titanium alloy, studying their connection mechanism and mechanical properties. However, liquid phase diffusion welding and laser welding processes are only suitable for local connections of plates and not for the overall composite connection of composite plates. Therefore, compared to explosive welding and diffusion welding methods, the rolling composite method has a stable product quality and simple equipment, and can easily achieve automation and large-scale production. In recent years, the rolling method has been widely studied due to its efficiency and economy and has been applied to the preparation of other homogeneous or heterogeneous metal laminated composite plates [18,19,20,21,22].

However, due to the significant difference in mechanical properties between titanium and magnesium, deformation coordination issues arise during hot rolling, and when the deformation is large, necking and fracture of the titanium layer occur, which has become a bottleneck in the preparation of Ti/Mg composite plates by rolling [23]. Therefore, a method of differential temperature rolling of Ti/Mg composite plates by separately heating the titanium plate with a resistance furnace was proposed [24], solving the problem of deformation coordination between titanium and magnesium, and achieving high bonding strength at a high reduction. This method was also successfully applied to the preparation of Ti/Al composite plates [25]. The downside is that the study found that the surface oxidation of the titanium plate was severe during the heating process of the differential temperature rolling, and the brittle oxide layer hindered the combination of fresh metal at a low reduction. Moreover, the presence of the brittle oxide layer has a negative impact on the tensile and bending properties of the composite plates.

To avoid the formation of an oxide layer on the surface of the plates after prolonged heating, which would greatly reduce the bonding performance of the composite plates, this work innovatively proposes a method of differential temperature rolling with longitudinal electromagnetic induction heating under a protective atmosphere. The principle mainly utilizes the thermal effect of eddy current heating, which can rapidly increase the temperature of ferromagnetic materials in a short period of time. A unique billet arrangement is designed, with pure iron plates used as the intermediate layer, taking advantage of the rapid temperature rise of the iron plate, and then transferring the heat sequentially to the titanium plates and the outermost magnesium plates, thus creating a significant plate temperature difference. This method can achieve the purpose of rapid heating of the billet while avoiding oxidation of the plates during the heating process and accurately controlling the temperature difference of the plates. Then, the effects of reduction on the bonding interface, shear strength, fracture morphology, and tensile delamination of the composite plates are studied in detail, the relationship between micromorphology and macroscopic mechanical properties is discussed, and the bonding mechanism of Ti/Mg composite plates is proposed.

## 2. Materials and Experimental Procedure

### 2.1. Materials Preparation

The experimental materials are 2 mm thick industrial pure titanium TA1, AZ31B magnesium alloy, and pure iron plates used as the intermediate layer. Rectangular plates with initial rolling direction dimensions of 100 mm × 60 mm are taken, and the chemical element content of the used TA1 and AZ31B magnesium alloy plates is listed in Table 1. The mechanical properties of TA1 and AZ31B plates after annealing are shown in Table 2. The surfaces of the titanium and magnesium plates to be combined are treated to remove surface oil, impurities, and oxides, which is conducive to the combination of fresh metal during rolling. In this experiment, a flat sanding machine equipped with 180-grit sandpaper (manufactured by Zhejiang Minli Power Tools Co., Ltd., Jinhua, China) is first used to remove impurities and oxides from the metal surfaces to be combined. Then, the surface is repeatedly wiped with acetone and alcohol and immediately dried with a hair dryer.

### 2.2. Differential Temperature Rolling Process with Induction Heating

#### 2.2.1. Billet Arrangement

**Figure 1:**
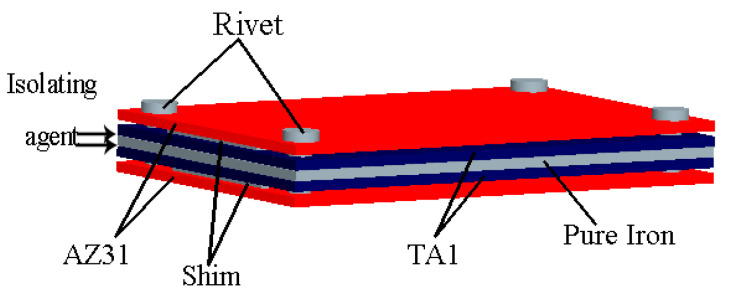
Schematic diagram for the symmetrical structure of multilayer plates.

The experimental design of the multilayer symmetrical billet arrangement is shown in Figure 1. The plates are stacked in a symmetrical multilayer structure in the order of Mg-Ti-pure iron-Ti-Mg. The advantage of the symmetrical structure is that it can not only make full use of the heat of the iron plate for energy saving, but also effectively reduce the warping deformation of the Ti/Mg composite plate after rolling. Since the thermal effect of eddy current heating can rapidly increase the temperature of ferromagnetic materials in a short time, a pure iron plate is chosen as the intermediate layer. Utilizing the rapid temperature rise of the iron plate, the heat is then sequentially transferred to the titanium plates and the outer magnesium plates, creating a significant temperature difference between the plates. To enable the separation of the iron plate and the Ti/Mg composite plate after rolling, talcum powder is selected as a lubricant between the iron and titanium plates. Additionally, spacers are placed on both sides of the titanium and magnesium plates to control the heat transfer rate between titanium and magnesium by leaving a gap of 0~1 mm. If the gap is too large, it will not be conducive to the plates biting into the rolling mill. Finally, the ends of the billet are drilled and fixed with aluminum rivets.

#### 2.2.2. Differential Temperature Rolling Process

**Figure 2:**
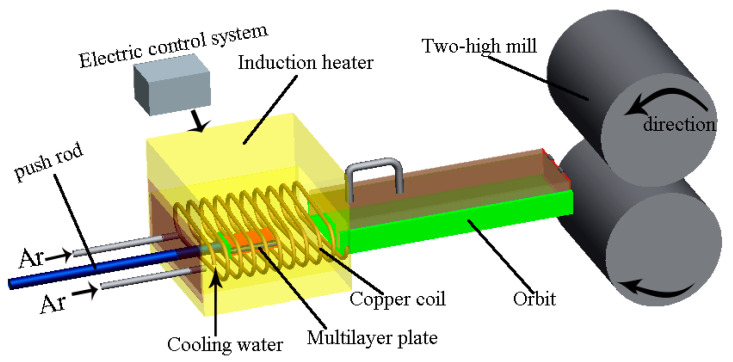
Schematic diagram of differential temperature rolling process with induction heating.

Figure 2 show the schematic diagram for the rolling process of Ti/Mg laminated composites by differential temperature rolling with induction heating. The induction heating equipment mainly consists of a spiral coil, a cooling system, and an electrical control system. The assembled multilayer billet is placed in the center of the induction heating furnace, and an appropriate induction current and heating time are applied. After the heating is completed, the billet is immediately pushed into the rolling mill for differential temperature rolling. The entire process from induction heating to rolling is sealed and protected by argon gas to prevent oxidation of the billet surface during heating. The mid-frequency induction heating furnace used in the experiment has a controllable induction current range of 0~2400 A.

#### 2.2.3. Determination of Rolling Process Parameters

**Figure 3:**
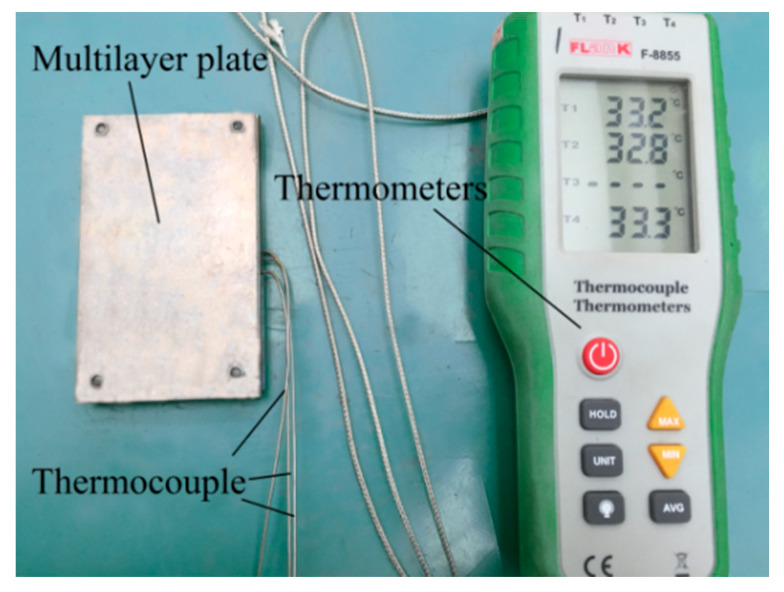
Actual temperature measurement procedure for the laminated plates.

**Figure 4:**
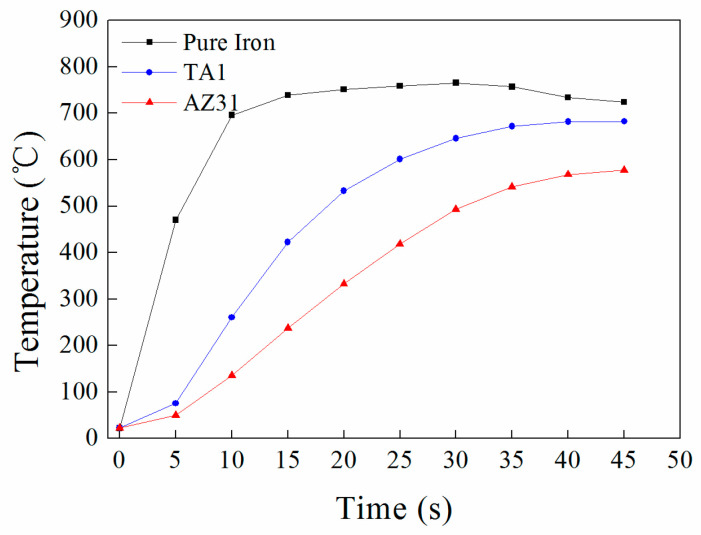
Temperature variation in individual laminated composite under 2100 A current and 1 mm clearance between TA1 and AZ31B.

In this experiment, a thermocouple thermometer (Flank F-8855) (Suzhou TASI Electronics Co., Ltd., Suzhou, China) is used to measure the temperature changes of the pure iron plate, titanium plate, and magnesium plate during the heating process. The actual temperature measurement device for the billet is shown in Figure 3. First, a hole with a diameter of 1 mm and a depth of 30 mm is drilled at the midpoint on the edge of the plates. Then, one end of the thermocouple wire (K-type, range −200 °C to 1372 °C, error ± 1 °C) is inserted into the hole, and the other end is connected to the thermometer. Figure 4 shows the temperature changes of each layer of the plate under a 2100 A induction current and a 1 mm gap between TA1 and AZ31B. After heating for 20 s, the temperature of the titanium plate is 532 °C, and the magnesium plate is 318 °C, with a maximum temperature difference of 214 °C. Therefore, in this experiment, a gap of 1 mm is chosen between the titanium and magnesium through aluminum spacers, a current of 2100 A is applied, and the plates are heated for 20 s before being immediately pushed into the rolling mill for rolling. The rolling reduction amounts are 17.5%, 32.5%, and 47.5%, all of which are single-pass reductions, and the reduction amounts are measured by the thickness of the titanium/aluminum composite plate after rolling. The parameters of the two-roll mill used in the experiment are: the roll size is φ200 mm × 200 mm, the rolling speed is 50 mm/s, and there is no lubrication.

### 2.3. Mechanical Properties Test and Microstructure Observation

**Figure 5:**
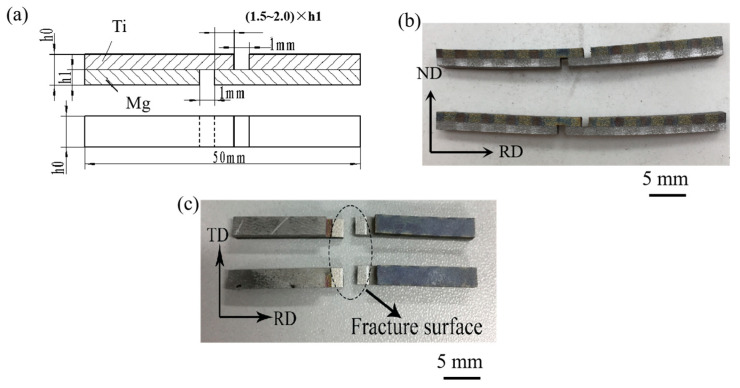
Tensile–shear test specimen and interface observation of the laminated composites: (**a**) schematic of the specimen; (**b**) real image of tensile–shear specimen; (**c**) fractured specimen.

**Figure 6:**
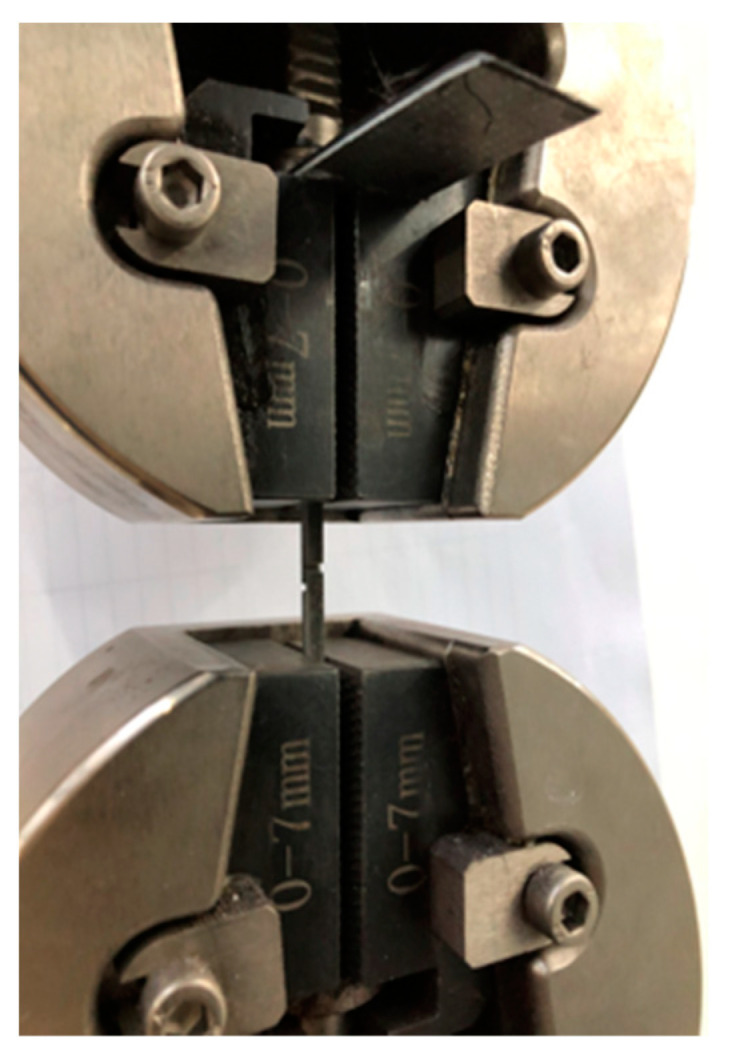
Actual tensile–shear testing process of specimen in the machine.

The bonding area ratio and the shear strength of the bimetal plates are two key parameters for the measurements of the properties of the laminated composites. The specimens for the tensile–shear test were made according to the GB/T 6396-2008 [26] (clad steel plates–mechanical and technological test) and GB/T 8547-2006 [27] (titanium clad steel plate) standards. Three specimens were selected from each plate parallel to the rolling direction and were tested to obtain the average shear strength, which was calculated according to the formula: bonding strength = peak loading/(bond width × bond length). Figure 5a–c show the geometry of the tensile–shear test specimen and fractured surface observed in the RD, TD and ND, respectively. The shear test was conducted at room temperature on an INSPEKT Table 100 kN electronic (Esum Technology Limited., Beijing, China) universal testing machine, with a shear rate of 1 mm/min, as shown in Figure 6. The tensile performance of the composite plate in the rolling direction is tested to evaluate the ductility, overall tensile strength, and the relationship between bonding performance and tensile performance.

Metallographic specimens are taken parallel to the rolling direction, and they are ground with sandpaper from 400# to 5000#, followed by rough polishing with a 2.5 um grit diamond polishing paste. Finally, a 1.5 um grit diamond polishing paste is used for fine polishing. The bonding interface and the morphology of the shear fracture are observed using an FEI Scios scanning electron microscope (SEM) (Thermo Fisher Scientic, Tokyo, Japan), and the elemental distribution near the bonding interface and the fracture is analyzed using an energy-dispersive spectrometer (EDS) (Thermo Fisher Scientic, Tokyo, Japan).

## 3. Results and Discussion

### 3.1. Macroscopic Bonding Property

**Figure 7:**
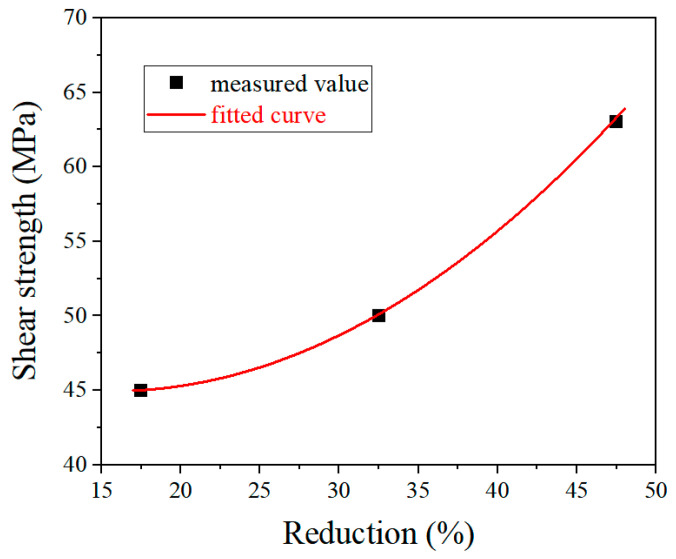
Effect of reduction on the shear strength of the Ti/Mg laminated composites.

Figure 7 shows the effect of reduction on the shear strength of the Ti/Mg laminated composites. Due to the symmetrical billet arrangement, the overall thickness of the plates is relatively large, and the rolling capacity of the mill is limited. According to the actual measured values, curve fitting is performed, and the fitted equation is y = 0.018x^2^ − 0.56x + 49.3, where x represents the value of the reduction, ranging from 17.5 to 47.5, and Y represents the shear strength. R^2^ is a statistical measure that assesses the discrepancy between the fitted curve and the actual data, with values ranging from 0 to 1. The closer the value is to 1, the closer the fitted curve is to the actual data; conversely, the further away it is. The R^2^ value of the curve fitted in this work is almost close to 1, indicating a good fit between the curve fitting and the measured values. The maximum single-pass reduction achieved for the Ti/Mg composite plate is 47.5%. Therefore, this experiment only compares the bonding strength of the composite plates at a similar reduction via a differential temperature rolling with the resistance furnace heating. From Figure 5, it can be seen that as the reduction increases, the shear strength of the composite plate gradually increases. At a reduction of 47.5%, the shear strength reaches a maximum of 63 MPa, which is nearly a 19% increase compared to the 53 MPa shear strength of the Ti/Mg composite plate at a 46% reduction using differential temperature rolling with resistance furnace heating [24]. More significantly, at a reduction of 17.5%, the shear strength reaches 45 MPa, which is nearly double the 25 MPa shear strength at a 25% reduction using differential temperature rolling with resistance furnace heating [24]. Therefore, the rapid induction heating combined with argon gas protection plays a key role in maintaining the cleanliness of the plate surface, avoiding the formation of oxides, thus allowing the fresh titanium and magnesium metals to come into contact at a low reduction, thereby significantly improving the interface bonding performance. Subsequently, the micro-interfaces and fracture morphologies of the Ti/Mg composite plates are characterized to further illustrate the microstructural features of the composite plates under this differential temperature rolling process.

### 3.2. Bonding Interface and Fracture Morphology

**Figure 8:**
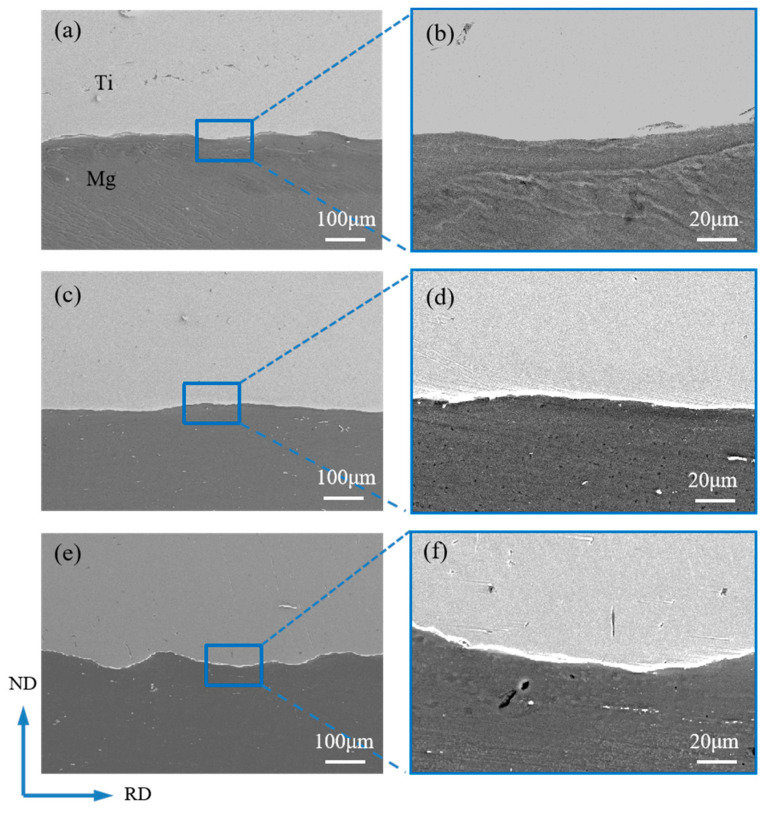
Bonding interface of laminated composites under different reductions: (**a**) 17.5% reduction; (**b**) enlarged area in image (**a**); (**c**) 32.5% reduction; (**d**) enlarged area in image (**c**); (**e**) 47.5% reduction; (**f**) enlarged area in image (**e**).

Figure 8 shows the SEM images of the bonding interface of the composite plates under different reductions. No obvious unbonded areas such as holes were observed at the bonding interface in the images, indicating that the titanium has undergone relatively large plastic deformation and has good fluidity, resulting in close contact at the interface and achieving a good composite effect. When the reductions are 17.5% and 32.5%, the Ti/Mg bonding interface appears relatively straight in both low and high magnifications, as shown in Figure 8a–d. As the reduction increases to 47.5%, the overall bonding interface in Figure 8e shows a distinct wavy shape, and the enlarged Figure 8f also reveals microscopic wavy interfaces. The change in the interface shape is consistent with the interface change in the differential temperature rolling with resistance furnace heating [24], mainly due to the large deformation rate of titanium at high temperatures in both processes, where the intense plastic deformation of the two metals on either side of the interface leads to the formation of waves. The wavy interface can withstand greater shear force during shearing, which is beneficial for improving the bonding strength of the composite plate. No obvious oxide layer was observed at the interface in Figure 8a–f, and the interface has always been in a clean state, which is different from the resistance furnace heating differential temperature rolling. The clean interface ensures the contact of fresh metals and enhances the inter-diffusion ability of elements, thereby overall improving the bonding performance of the composite plate.

**Figure 9:**
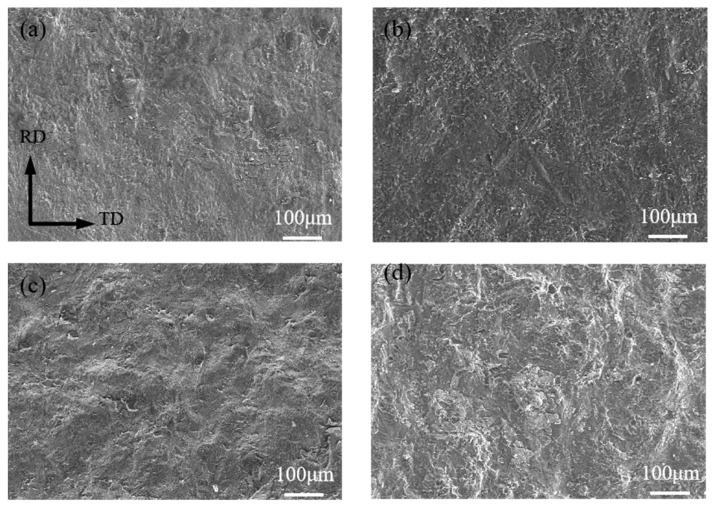
Overall morphology of the tensile–shear fracture of composite plates: (**a**) 32.5%/Ti side; (**b**) 32.5%/Mg side, (**c**) 47.5%/Ti side, (**d**) 47.5%/Mg side.

Figure 9 presents the overall morphology of the tensile–shear fracture of the composite plates under different reductions. As can be seen from Figure 8c,d, at a 32.5% reduction, the bonding interface is straight, and after the shear test, the fracture surfaces on the titanium and magnesium sides are shown in Figure 9a and Figure 9b, respectively. Macroscopically, both fracture surfaces appear relatively flat. At a higher reduction of 47.5%, the fracture surfaces on both the titanium and magnesium sides, as shown in Figure 9c,d, exhibit distinct wavy characteristics, which correspond to the morphology of the bonding interface. To further observe the finer microstructures at the fracture, the fracture surfaces were magnified and subjected to EDS elemental scanning tests.

**Figure 10:**
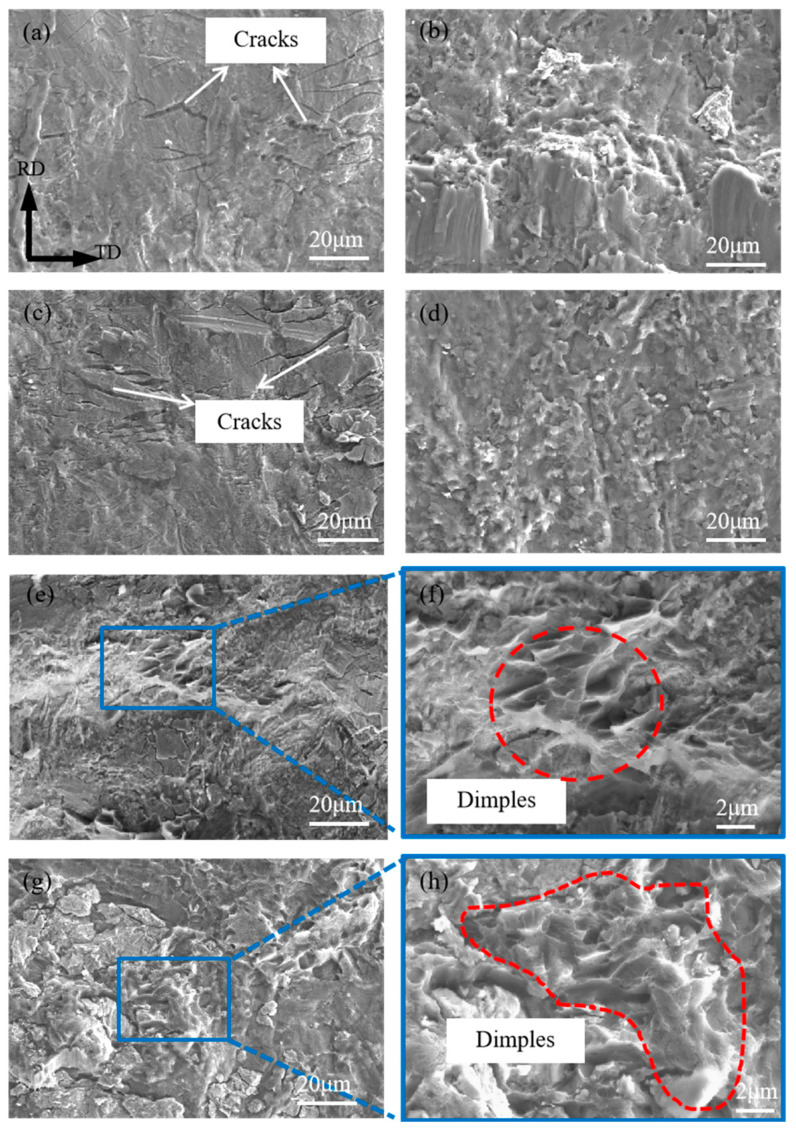
Microscopic morphology of the tensile–shear fracture of composite plates: (**a**) 17.5%/Ti side; (**b**) 17.5%/Mg side; (**c**) 32.5%/Ti side; (**d**) 32.5%/Mg side; (**e**) 47.5%/Ti side; (**f**) enlarged area; (**g**) 47.5%/Mg side; (**h**) enlarged area.

**Figure 11:**
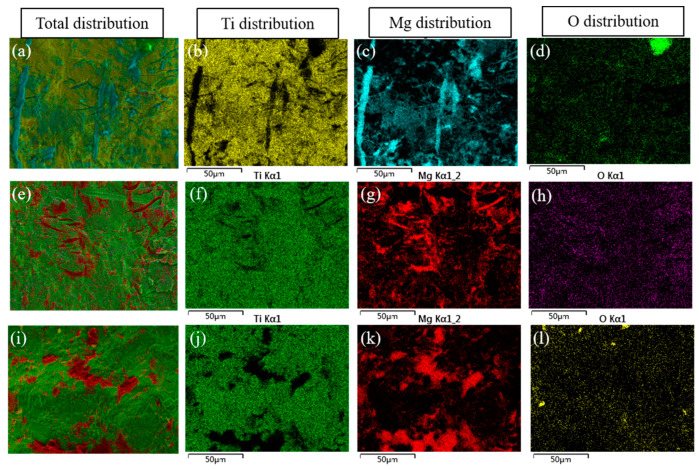
Element mapping scanning in Ti side of the tensile–shear fracture under different processes: (**a**) 17.5%; (**b**–**d**) Ti, Mg and O distributions in image (**a**); (**e**) 32.5%; (**f**–**h**) Ti, Mg and O distributions in image (**e**); (**i**) 47.5%; (**j**–**l**) Ti, Mg and O distributions in image (**i**).

Figure 10 shows the microscopic morphology of the tensile–shear fracture of the composite plates, and Figure 11 presents the EDS elemental scanning results of the titanium side fracture under different reduction rates. Figure 10a,c show that at reduction rates of 17.5% and 32.5%, vertical cracks are produced on the titanium side, perpendicular to the rolling direction, and the fracture surface is characteristic of typical brittle fracture. Additionally, from the elemental scanning results in Figure 11a–h, it can be seen that the cracks are filled with Mg elements. Moreover, apart from the crack areas, the titanium side also contains some Mg elements, indicating that the titanium side has adhered to some magnesium metal, with evidence of magnesium metal squeezing into the titanium cracks. Comparing the distribution of O elements with that of Ti and Mg elements at the fracture, it is found that the distribution of O is random and does not follow any discernible pattern related to the distribution of Ti and Mg, suggesting that the presence of oxygen is not due to oxidation during the heating and rolling processes of the plates, but rather due to oxidation that occurs when the fracture is exposed to air after shearing. Figure 10b,d show that at reductions of 17.5% and 32.5%, the magnesium side also exhibits characteristics of brittle fracture, suggesting that at low reductions, the fracture of the composite plate primarily occurs at the interface between titanium and magnesium. As the reduction increases to 47.5%, the fracture on the titanium side, as shown in Figure 10e,f, is undulating with the formation of some dimples. The EDS scanning test results in Figure 11i–l show that all the dimples on the titanium side are filled with Mg elements, while the remaining non-dimple areas are filled with Ti elements, indicating that the formation of some dimples is due to the fracture of the magnesium matrix. Correspondingly, dimples are also formed on the magnesium side, as shown in Figure 10g,h. Therefore, it is inferred that at a higher reduction, the fracture of the composite plate occurs at a mixed location between the magnesium matrix and the Ti/Mg bonding surface. The proportion of dimples on the entire fracture surface suggests that there is still significant room for improvement in the Ti/Mg composite plate.

### 3.3. Tensile Property and Interfacial Delamination of Laminated Composites

**Figure 12:**
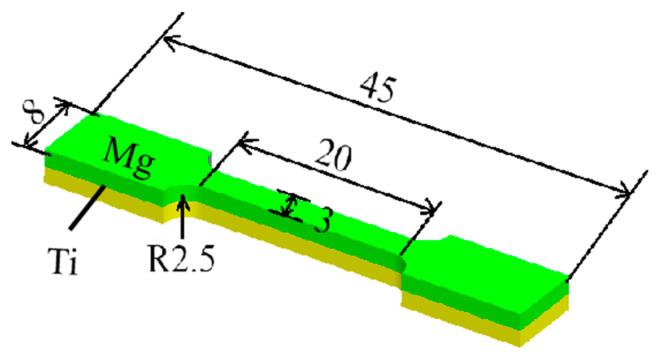
Schematic diagram of the tensile specimen (distances are in mm).

**Figure 13:**
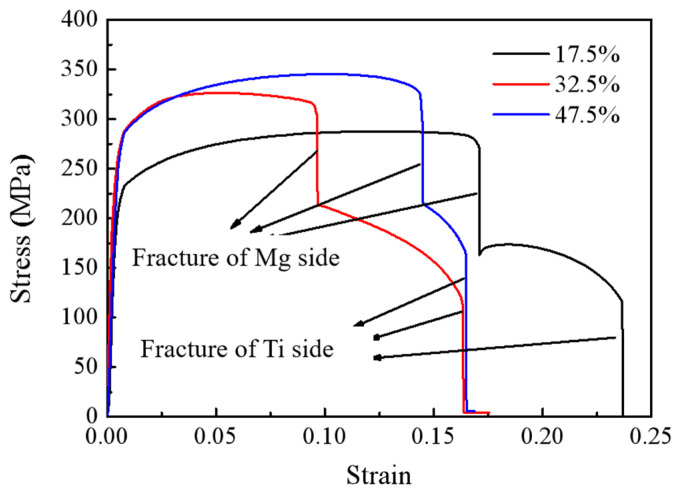
Engineering stress–strain curves of Ti/Mg laminated composites.

**Figure 14:**
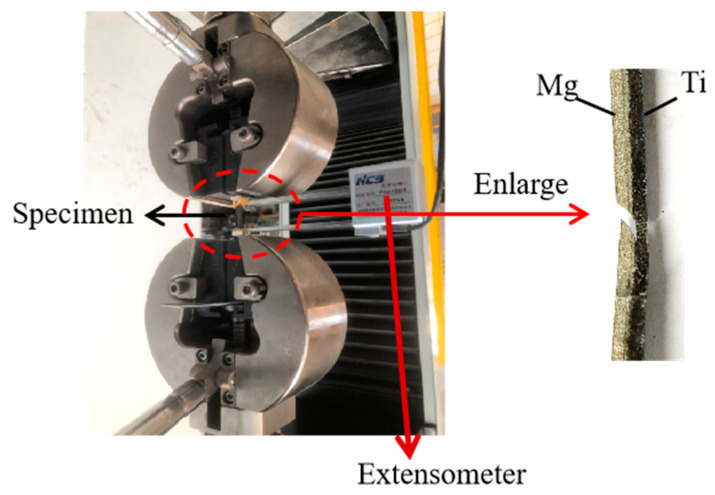
The tensile fracture process of Ti/Mg laminated composite.

The schematic diagram of the tensile specimen is shown in Figure 12. Figure 13 presents the overall tensile performance test of the Ti/Mg composite plates, and the engineering stress–strain curves are shown in the figure. At a reduction of 17.5%, the overall tensile strength of the composite plate is about 300 MPa. As the rolling reduction increases, the tensile strength gradually increases, reaching a maximum of about 350 MPa at a 47.5% reduction. This is because, with the increase in the reduction rate, both the titanium and magnesium layers in the composite plate undergo work hardening, resulting in an increase in the strength of each layer, which in turn increases the overall tensile strength of the composite plate. Since the elongation rate of the magnesium plate is much lower than that of the titanium plate, during the tensile process, it is observed that the magnesium plate breaks first, followed by the continued plastic deformation and subsequent fracture of the titanium plate, as shown in Figure 14. This leads to the noticeable step-like drops in the three tensile curves in Figure 12. The first vertical drop is due to the fracture of the magnesium plate, and the second vertical drop is due to the eventual fracture of the titanium plate. Due to the effect of work hardening, while the tensile strength is improved, the total elongation rate of the composite plate shows a downward trend. However, the tensile curves show that the overall elongation rate of the composite plates at reductions of 32.5% and 47.5% is essentially the same. It is analyzed that this may be due to the increased bonding strength of the composite plate, which promotes the coordinated deformation of the titanium and magnesium plates during the overall tensile process, resulting in the composite plate at a 47.5% reduction having the same elongation rate as the composite plate at a 32.5% reduction.

**Figure 15:**
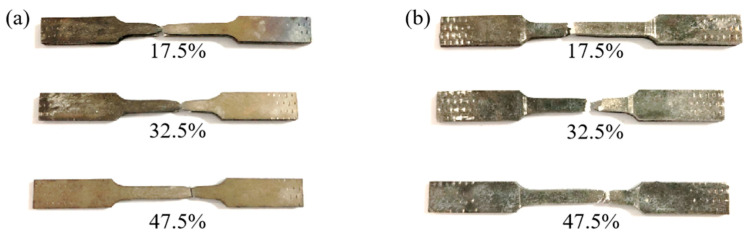
Tensile fracture of Ti/Mg laminated composites: (**a**) Ti side; (**b**) Mg side.

After the tensile fracture of the composite plate, the fracture surfaces were spliced together, and the macroscopic appearance of the titanium and magnesium sides after splicing is shown in Figure 15. It can be seen from Figure 15a that under different reductions, the composite plate ultimately fails with the fracture of the titanium plate, and a significant necking phenomenon occurs on the titanium plate. Figure 15b shows that the elongation rate decreases significantly from a 17.5% to a 32.5% reduction rate, and the elongation rate is relatively consistent from a 32.5% to a 47.5% reduction. However, the fracture length of the magnesium side of the composite plate is significantly larger at a 32.5% reduction, indicating that a weaker bonding interface does not play a restraining role on the fracture of the two plates during the tensile process, while a stronger bonding interface allows the titanium plate to pull the magnesium plate, preventing the premature fracture of the magnesium plate and achieving more consistent coordinated deformation.

**Figure 16:**
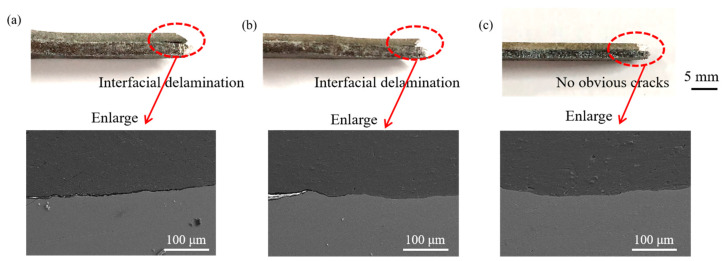
Interfacial delamination of the tensile fracture: (**a**) 17.5%; (**b**) 32.5%: (**c**) 47.5%.

Figure 16 shows the interfacial delamination situation of the tensile fracture. Figure 16a shows that at a 17.5% reduction, the composite plate has the longest interface crack propagation and obvious interfacial delamination. As the reduction increases and the bonding strength increases, the interfacial delamination is gradually improved, as shown in Figure 16b. At a 32.5% reduction, the length of the interface crack in the composite plate becomes shorter, and at a 47.5% reduction, no obvious macroscopic cracks are observed in Figure 16c, indicating that the composite plate has obtained better resistance to interfacial delamination. The reason for the improved resistance to interfacial delamination is analyzed as follows: during the tensile process, the plates undergo necking, and the different necking tendencies of titanium and magnesium will generate shear force at the interface. A weak bonding interface cannot resist this shear force, leading to interface cracking and subsequent crack propagation. However, a strong bonding interface can resist this shear force, allowing the two metals to have the same necking tendency, thus avoiding interfacial delamination of the composite plate.

### 3.4. Bonding Mechanism

**Figure 17:**
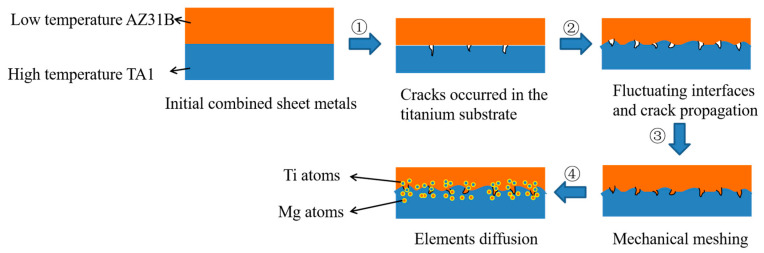
Schematic diagram of Ti/Mg bonding mechanism. (1) cracks occurred in the titanium substrate, (2) fluctuating interfaces and crack propagation, (3) mechanical meshing, (4) elements diffusion.

Figure 17 presents a schematic diagram of the Ti/Mg bonding mechanism. Based on the above analysis of the tensile–shear fracture surface and the micro-morphology of the composite interface, the bonding mechanism of the differential temperature rolled Ti/Mg composite plate with induction heating is summarized, as shown in the diagram. In the initial stage of low reduction rolling, the titanium matrix, due to its larger plastic deformation, first produces micro-cracks. As the reduction increases, the bonding interface gradually exhibits a typical wavy appearance, accompanied by crack characteristics on the titanium side. When the reduction is further increased, the magnesium side metal squeezes into the cracks of the wavy interface, forming mechanical interlocking. Finally, under the action of pressure and temperature, mutual diffusion of titanium and magnesium elements occurs, achieving a better bonding performance. The difference in the bonding mechanism of the differential temperature rolled Ti/Mg composite plate with induction heating compared to the resistance furnace heating is that there is no oxide formation at the titanium matrix interface, which promotes full contact of fresh metals and mutual diffusion of elements, ultimately achieving the effect of improving the bonding strength of the composite plate.

## 4. Conclusions

This work has innovated a method for preparing Ti/Mg metal laminated composites by differential temperature rolling with longitudinal electromagnetic induction heating under a protective atmosphere. This method successfully avoids oxidation of the plates during the rapid heating of the billet and has studied the effects of reduction and the presence or absence of an oxide layer on the bonding interface, shear strength, fracture morphology, and tensile interface delamination of the two types of composite plates. It has also explored the relationship between micro-morphology and macroscopic mechanical properties. The detailed conclusions are as follows:

(1) A clean and oxide-free bonding interface promotes contact between fresh metals and mutual diffusion of elements, which improves the bonding strength of the double-layer composite plates compared to those made by resistance furnace heating. Especially, it significantly enhances the bonding strength of the composite plates at a low reduction.
(2) With the increase in bonding strength, the fracture surfaces on both sides of the shear fracture gradually change from brittle fracture to ductile fracture. This is attributed to the fact that at low bonding strengths, the shear fracture occurs at the interface between the two plates, while at high bonding strengths, the shear fracture occurs within the metal matrix.
(3) A strong bonding interface is beneficial in preventing the propagation of interfacial cracks during the tensile testing of Ti/Mg composite plates, thereby enhancing the composite plates’ ability to resist interfacial delamination.
(4) The bonding interface of the Ti/Mg composite plates prepared by differential temperature rolling with induction heating is primarily achieved through a combination of mechanical interlocking and elemental diffusion.

## Figures and Tables

**Table 1:** Chemical composition of commercial pure Ti-TA1 sheet, Mg alloy-AZ31B sheet (wt%).

Materials	C	N	H	O	Si	Fe	Al	Ca	Zn	Mn	Cu	Ti	Mg
TA1	0.05	0.03	0.015	0.15	0.1	0.15	-	-	-	-	-	Bal.	-
AZ31B	-	-	-	-	0.08	0.03	3.1	0.04	0.9	0.5	0.01	0.15	Bal.

**Table 2:** Mechanical properties of the used materials in the experiment.

Materials	Ultimate Tensile Strength (MPa)	Yield Strength (MPa)	Shear Strength (MPa)	Fracture Elongation (%)
TA1	434	328	285	25.3
AZ31B	226	160	128	12.5

## Data Availability

The raw data supporting the conclusions of this article will be made available by the authors on request.
